# Functional and Structural Alterations in the Cingulate Motor Area Relate to Decreased Fronto-Striatal Coupling in Major Depressive Disorder with Psychomotor Disturbances

**DOI:** 10.3389/fpsyt.2014.00176

**Published:** 2014-12-04

**Authors:** Benny Liberg, Paul Klauser, Ian H. Harding, Mats Adler, Christoffer Rahm, Johan Lundberg, Thomas Masterman, Caroline Wachtler, Tomas Jonsson, Maria Kristoffersen-Wiberg, Christos Pantelis, Björn Wahlund

**Affiliations:** ^1^Department of Psychiatry, Melbourne Neuropsychiatry Centre, The University of Melbourne, Melbourne, VIC, Australia; ^2^Section of Psychiatry, Department of Clinical Neuroscience, Karolinska Institutet, Stockholm, Sweden; ^3^Department of Clinical Science, Intervention and Technology (CLINTEC), Division of Medical Imaging and Technology, Karolinska Institutet, Stockholm, Sweden; ^4^Monash Clinical and Imaging Neuroscience, School of Psychology and Psychiatry, Monash University, Clayton, VIC, Australia; ^5^School of Psychological Sciences, Monash University, Melbourne, VIC, Australia; ^6^Department of Medicine Huddinge, Karolinska Institutet, Stockholm, Sweden; ^7^Primary Care Research Unit, Department of General Practice, The University of Melbourne, Melbourne, VIC, Australia; ^8^Centre for Family Medicine (CeFAM), Department of Neurobiology, Care Sciences and Society, Karolinska Institutet, Stockholm, Sweden; ^9^Department of Medical Physics, Karolinska University Hospital Huddinge, Stockholm, Sweden; ^10^Department of Radiology, Karolinska University Hospital, Stockholm, Sweden; ^11^Department of Energy and Engineering, Swedish University of Agricultural Sciences, Uppsala, Sweden

**Keywords:** major depression, bipolar disorder, psychomotor disturbances, cingulate cortex, cingulate motor area, striatum, putamen

## Abstract

Psychomotor disturbances are a classic feature of major depressive disorders. These can manifest as lack of facial expressions and decreased speech production, reduced body posture and mobility, and slowed voluntary movement. The neural correlates of psychomotor disturbances in depression are poorly understood but it has been suggested that outputs from the cingulate motor area (CMA) to striatal motor regions, including the putamen, could be involved. We used functional and structural magnetic resonance imaging to conduct a region-of-interest analysis to test the hypotheses that neural activation patterns related to motor production and gray matter volumes in the CMA would be different between depressed subjects displaying psychomotor disturbances (*n* = 13) and matched healthy controls (*n* = 13). In addition, we conducted a psychophysiological interaction analysis to assess the functional coupling related to self-paced finger-tapping between the caudal CMA and the posterior putamen in patients compared to controls. We found a cluster of increased neural activation, adjacent to a cluster of decreased gray matter volume in the caudal CMA in patients compared to controls. The functional coupling between the left caudal CMA and the left putamen during finger-tapping task performance was additionally decreased in patients compared to controls. In addition, the strength of the functional coupling between the left caudal CMA and the left putamen was negatively correlated with the severity of psychomotor disturbances in the patient group. In conclusion, we found converging evidence for involvement of the caudal CMA and putamen in the generation of psychomotor disturbances in depression.

## Introduction

Psychomotor disturbance is a cardinal feature of major depressive disorder. The severity of psychomotor disruption is clinically associated with depression severity and predicts response to certain pharmacological treatments (1–4). Psychomotor disturbances transcend illness phase and manifest as changes in interactiveness and spontaneity, alongside reductions in facial expression, body mobility, postural tone, speed of movement, and speech (5–7). Investigations of the neural correlates underpinning psychomotor disturbances remain sparse; indeed, the motor system has been relatively neglected in brain imaging studies of psychiatric disorders in general (8). However, available evidence points to metabolic deficits, neurochemical changes, and altered structural connections and functional connectivity involving large-scale brain networks that connect frontal cortical regions and subcortical (esp. basal ganglia) areas (9–24). Interactions between the striatum, frontal motor regions, and prefrontal association cortices are known to be critical to the initiation and regulation of motor output and cognitive processing (25, 26). However, the localization and relevance of brain abnormalities in these fronto-striatal systems to psychomotor disturbances observed in depression remains largely unknown.

One important target for investigation of psychomotor disturbances is the cingulate cortex. Converging evidence supporting the involvement of the cingulate cortex in the clinical expression of major depressive disorder comes through experimental studies using interventions, such as sadness induction (27), cognitive behavioral therapy (28), pharmacological probes, such as antidepressants and dopamine-modulating treatments (29, 30), or neuromodulation such as electroconvulsive therapy or transcranial magnetic stimulation (31–35). Additionally, secondary cingulate cortex lesions can also lead to a neuropsychiatric condition known as akinetic mutism that involves severe alterations of volition, psychomotor slowing, and apathy – clinical features that resemble severe major depressive disorder (36, 37).

The cingulate cortex has been suggested to integrate volition, affect, and behavior. It is located on the midline rim of the corpus callosum and is generally divided anatomically into four distinct subregions (38, 39). One of these subregions is the midcingulate region, which contains the cingulate motor area (CMA) (40, 41). The CMA is a cortical midline structure, located in the posterior frontal lobe, superior to the corpus callosum, and inferior to the supplementary motor area. The CMA has been implicated in motor behaviors with affective incentives (42) and receives neural signals from affective limbic regions, frontal executive regions, and motor regions (37, 43). A caudal subregion of the CMA has long been implicated in the execution of simple motor tasks, but only recently has its somatotopy and functional organization been determined with more spatially precise brain imaging (44–47). Functional connectivity and retrograde tracings in primates suggest inputs from caudal CMA to lateral putamen in general, and to rostral ventral putamen in particular, overlap with inputs from primary motor areas (48, 49).

There is currently no empirically validated functional anatomical model of psychomotor disturbances in major depressive disorder. However, within the anatomical framework of large-scale brain networks, Vogt proposed one such pathophysiological model in which a loss of neurons in the cortical output layer (layer V) of the cingulate cortex specifically attenuates the output from the CMA to subcortical motor regions, resulting in a paucity of internally guided movement and speech (50). Clinically, this neural disruption could be reflected in the altered response to external events and slowed movements that characterize psychomotor disturbances observed in major depressive disorder.

Using the framework of Vogt’s theory of movement paucity in major depressive disorder, we hypothesized that psychomotor disturbances in major depressive disorder rely on structural and functional abnormalities in a network encompassing the CMA and striatal motor regions. We predict that participants with major depressive disorder show modifications of functional activation in left CMA and left posterior putamen during a task involving the generation of rapid right-lateralized finger movements (i.e., self-paced finger-tapping). We also anticipate that these functional alterations are accompanied by modifications of gray matter volume in both the left CMA and left putamen. Finally, the severity of both functional and structural abnormalities is predicted to correlate with the degree of observed psychomotor disturbances.

## Materials and Methods

### Study sample

The Karolinska University Hospital and Stockholm City Council Ethics Committee approved the study protocol. Each subject gave oral and written informed consent to participate in this study. Thirteen patients with a bipolar I diagnosis (*n* = 9), bipolar II diagnosis (*n* = 1), or unipolar depression diagnosis (*n* = 3) were recruited from The Affective Disorders Unit at Psychiatry Southwest at Karolinska University Hospital in Huddinge, Sweden. All patients were experiencing a current episode of depression with a duration > 1 month and featuring psychomotor disturbances. No patient fulfilled the diagnostic criteria for concurrent mania, hypomania, or rapid cycling disorder. Thirteen healthy controls without psychiatric diagnoses were recruited. Clinical diagnoses were confirmed using a computerized version of the Structured Clinical Interview for DSM Disorders (51). All participants were right handed, had no history of neurologic illness, and had normal or corrected-to-normal visual acuity (52). All patients were on medication (Table 1), and all controls were drug free. Depression severity was rated by Benny Liberg and Mats Adler with the Montgomery Åsberg Depression Rating Scale (53) and psychomotor disturbance was rated with the CORE scale (54). Retrospective assessment of patient files did not reveal the presence of extrapyramidal symptoms or signs in medicated patients.

**Table 1:** **Sample characteristics**.

Variable	Controls	Patients
Sex	6 F, 7 M; *n* = 13	9 F, 4 M; *n* = 13
Age (years)	39 (29–67)	44 (24–62)
Bipolar depression (type I, type II)		*n* = 10 (*n* = 9, *n* = 1)
Unipolar depression (recurrent, first episode)		*n* = 3 (*n* = 2, *n* = 1)
MADRS total score		28.9 (11–48)
CORE total score		17.7 (10–36)
CORE retardation items score		9.1 (3–15)
AS-18 retardation factor score		8.2 (0–12)
AS-18 depression factor score		23.2 (1–36)
AS-18 mania factor score		4.9 (0–13)
AS-18 total score		36.2 (2–58)
Lithium		*n* = 4
Typical neuroleptics (FGA)		*n* = 2
Atypical neuroleptics (SGA)		*n* = 4
Anticonvulsants		*n* = 5
Antidepressant (TCA)		*n* = 1
Antidepressant (SSRI)		*n* = 1
Antidepressant (SNRI)		*n* = 5
MAO-I		*n* = 1
Thyroxine		*n* = 1
Electroconvulsive treatment		*n* = 2

*If not otherwise specified, values represent the mean and the range is given into brackets*.

*M/F, male/female; FGA, first-generation antipsychotics; SGA, second-generation antipsychotics; TCA, tricyclic antidepressants; SSRI, serotonin reuptake inhibitors; SNRI, serotonin-noradrenalin reuptake inhibitors; MAO-I, monoamine-oxidase inhibitors*.

#### Image acquisition

Participants performed finger-tapping while in the MRI-scanner, following instructions on a computer screen viewed through a head-coil mounted mirror. Before the experiment started, participants were shown how to perform finger-tapping, defined as a thumb-index finger opposition of the right hand. Participants were asked to tap as quickly as possible. The experiment had an ON/OFF design consisting of 20 s of finger-tapping followed by 20 s of rest. During the first second of each ON-period, the instruction “tap” was presented, and the screen then turned black for 19 s. Rest periods were indicated by a fixation-cross presented for 20 s. This stimulus cycle was repeated seven times. The total functional scanning time was 280 s.

A Siemens Avanto 1.5-T scanner was used to acquire blood oxygen level dependent (BOLD) sensitive T2*-weighted echo planar images. Each echo planar image comprised 22 axial slices with a resolution of 3.75 mm × 3.75 mm × 5 mm and an interslice interval of 1 mm. Volumes were acquired with a repetition time (TR) of 2.5 s, an echo time (TE) of 30 ms, a field-of-view of 64 mm × 64 mm, and a flip angle of 90°. The first six (dummy) volumes of each run were discarded to allow for T1 equilibration effects. A total of 112 volumes were acquired. After the functional scans had been collected, a T1-weighted anatomical image [magnetization prepared rapid acquisition gradient echo (MP-RAGE)], 128 slices; TR, 2400 ms; TE, 3.44 ms; with a voxel size of 1.3 mm × 1.3 mm × 1.3 mm] was acquired for all subjects. To rule out radiological signs of pathology, a consultant in neuroradiology (Maria Kristoffersen-Wiberg) assessed the anatomical scans of each subject.

#### Region-of-interest masks

We used the software GingerALE 2.3 to define the left caudal CMA mask. GingerALE allows for meta-analysis of human brain imaging studies using published co-ordinates in standard space (55). Co-ordinates were derived from a study that mapped the functional anatomy of the CMA at the single-subject level (44). Input foci data included co-ordinates in the left hemisphere that represented neural activation in the CMA during motor execution using the right hand. Significant clusters in the whole-brain analysis were determined by a (corrected using a Monte-Carlo approach) cluster significance threshold of *p* ≤ 0.05. The resulting ROI mask is illustrated in Figure 3. The ROI mask for the putamen region connecting to the caudal motor cortex was derived from the Oxford Imanova Striatal Connectivity Atlas (seven-regions) supplied with FSL (56).

#### Analysis of functional activations

Imaging data were analyzed using the FSL 5.0.2 software suite [The Oxford Centre for Functional MRI of the Brain (FMRIB), Oxford University, United Kingdom]. Data processing was carried out using the fMRI Expert Analysis Tool version 5.98. Rigid-body head motion correction was first performed (57). We subsequently de-noised the data using the multivariate ICA-based classifier FIX (58, 59), based on a conservative threshold of five components. Non-brain tissue was then removed (60) and the functional data were smoothed using a Gaussian kernel set to a full-width half-maximum (FWHM) of 6 mm. To account for time differences in slice acquisition, we performed slice-timing correction using Fourier-space time series phase shifting. In addition, we normalized the grand-mean intensity of the entire four-dimensional (4D) dataset by a single multiplicative factor and filtered out physiological noise using a high-pass temporal filter set to a period of 100 s. Registration of the data to standard anatomical space was undertaken with the high-resolution structural (T1) scan using boundary-based registration with BBR, and affine linear registration with FLIRT (61). Estimated transformations were subsequently applied to the co-registered functional data. The time series of each subject was modeled using a general linear model (GLM) containing a single predictor representing the on–off time-course of the experiment, convolved with a hemodynamic response function (gamma). Parameter estimates (PEs) were calculated for all brain voxels. Correction for local autocorrelation in the time series was undertaken using FILM (62).

The subject-specific contrast (COPE) images of the finger-tapping effect were then entered into a second-level group analysis. As our patient sample had a different sex distribution than our control group, we added age and sex as covariates in the regression model to remove their potential confounding effects. We also created a 4D covariate image of gray matter using the feat_gm_prepare script. The 4D image output containing gray matter partial volume information was inserted as voxel-dependent EVs in the GLM model for each subject (63). The higher level analysis was carried out using FMRIB’s local analysis of mixed effects stage 1 and stage 2 (64–66). *Z* (Gaussianized T/F) statistic images in the ROI analysis were thresholded using clusters determined by *Z* ≥ 2.3 and a (corrected using Gaussian random field theory) cluster significance threshold of *p* ≤ 0.05.

#### Analysis of functional connectivity

We performed a psychophysiological interaction (PPI) analysis to determine group differences in task-dependent alterations of functional coupling between the left caudal CMA and left putamen regions connected to caudal cortical motor regions. Analysis was performed in SPM8 using the preprocessed first-level data described above (preprocessed in FSL). A PPI analysis determines how the statistical dependency between the time courses of neural activation in a region-of-interest (ROI) and a targeted brain region depends on a task context (67). We extracted the BOLD time series data from each subject within a 5 mm sphere centered at the peak of the task-related group activation difference within the left CMA (see Results). At the first level of analysis, a GLM consisting of three predictors was specified: the left CMA time series data as a physiological regressor, the task-based model as psychological regressor, and the interaction term (the PPI) formed by their crossproduct. We entered first-level COPE images representing the interaction term into a second-level random effects analysis of group differences in a region of the left putamen connected to caudal cortical motor areas involved in movement. Inference was undertaken using two-sample *T*-tests restricted to a mask of the left putamen and corrected for multiple comparisons (*p* ≤ 0.05) based on minimum cluster-extent thresholds estimated using the AlphaSim permutation procedure (REST toolbox; http://pub.restfmri.net). Simulations were run using an uncorrected voxel-level threshold of *p* ≤ 0.05, across 1000 permutations, resulting in a minimum required cluster-threshold of 14 voxels (*p* ≤ 0.05, corrected at the mask level).

#### Analysis of gray matter volume

Paul Klauser and Benny Liberg inspected every image to assess the presence of artifacts or gross anatomical abnormalities that could impact image preprocessing. We estimated gray matter volume using voxel-based morphometry (VBM) implemented in SPM8 (http://www.fil.ion.ucl.ac.uk/spm/software/spm8/). Each participant’s T1-weighted anatomical scan was segmented into gray, white, and cerebrospinal fluid compartments using the VBM8 toolbox (http://dbm.neuro.uni-jena.de/vbm) set to default parameters. Native-space gray matter images were then spatially normalized to the DARTEL template in MNI standard space created from 550 healthy control subjects from the IXI-database (http://www.brain-development.org). For the generation of gray matter volumes, Jacobian determinants were used to modulate gray voxel intensities with non-linear warping only in order to preserve original gray matter volumes while discarding initial differences in brain sizes. The images were then smoothed with a 6 mm full-width-half-maximum Gaussian kernel prior to statistical analysis. A GLM was used to test for group differences in gray matter volume at each voxel within the CMA ROI mask, as implemented in Randomise (http://fsl.fmrib.ox.ac.uk/fsl/randomise). All results were corrected for multiple comparison type I error at the ROI mask level using a non-parametric cluster size-based procedure. We set the voxelwise cluster-forming threshold to *T* ≥ 2.5. Then, a clusterwise *p*-value corrected at the ROI level was calculated from a permutation test (10,000 permutations). Age and gender were entered as covariates in the GLM.

#### Correlation analyses

Calculation of Spearman’s Rho correlation coefficients between CORE ratings, imaging metrics, and neurobiological indices were assessed to determine associations between clinical phenomenology and brain abnormalities determined with different imaging modalities.

## Results

### Functional activation

**Figure 1:**
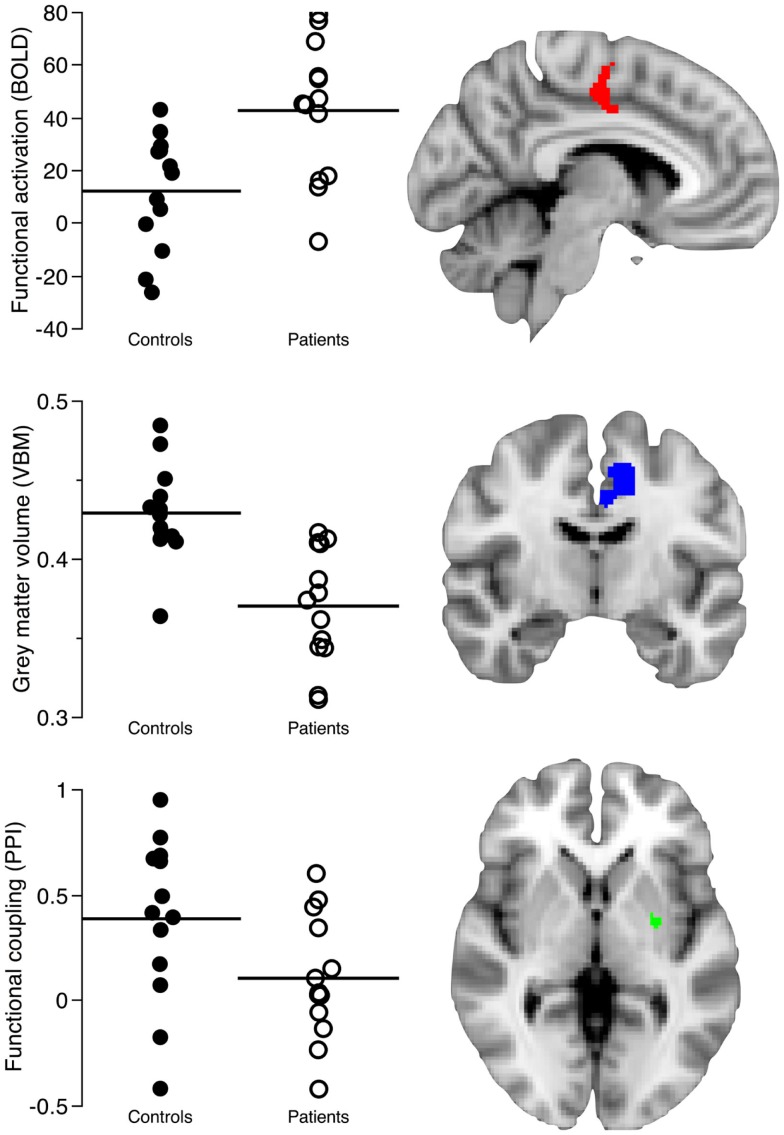
**The image shows the structural and functional imaging findings in the left caudal cingulate motor area (CMA) and putamen regions connected to caudal motor regions in the frontal lobe**. The first row shows the contrast parameter estimates reflecting how patients activate the caudal cingulate motor regions more than healthy controls (*Z* > 2.3, *p* = 0.05, corrected) during self-paced finger-tapping. The second row shows decreased gray matter volume in the left CMA of patients (*T* > 2.5, *p* = 0.05, corrected). The third row shows the decreased functional coupling of the left CMA and left posterior putamen regions connected to caudal motor regions in the frontal lobe during self-paced finger-tapping (*p* = 0.02, corrected). Metrics (mean) from clusters of between-group differences in the left caudal CMA and posterior putamen were retrieved using masks comprising 356 voxels/2848 mm^3^ from the functional magnetic resonance imaging analysis, 941 voxels/3176 mm^3^ from the voxel-based morphometry analysis, and 31 voxels/248 mm^3^ from the psychophysiological interaction analysis.

In our group, comparison of task-related neural activation in the caudal CMA during self-paced finger-tapping, we found that patients activated the left caudal CMA more than healthy controls (cluster size: 356 voxels/2848 mm^3^; *Z*_CMA_ = 3.01; *p* = 0.003, corrected at the cluster level; peak voxel: *x* = −6, *y* = −10, *z* = 38; Figure 1; Table 2).

**Table 2:** **Functional activation statistics during self-paced finger-tapping (patient > controls)**.

*Z*-max	Cluster	MNI co-ordinates			Anatomical label
		*x*	*y*	*z*	
3.85	1	2	−14	52	38% precentral gyrus, 36% SMA
3.66		−2	−14	50	38% SMA, 34% precentral gyrus
3.01		−6	−10	38	32% cingulate gyrus, anterior division (CMA)
2.75		4	−2	52	73% SMA
2.53		8	−2	48	49% SMA

### Functional connectivity

In our PPI analysis of task-related functional coupling between the caudal CMA and the posterior putamen regions connected to caudal motor regions in the frontal lobe, we found significant group differences (cluster size: 31 voxels/248 mm^3^, *p* = 0.02, corrected; peak voxel: *x* = −26, *y* = −6, *z* = 0; Figure 1). We found that neural activation in controls showed a task-related correspondence between the left caudal CMA and left posterior putamen regions connected to caudal motor regions in the frontal lobe, whereas this relationship was absent in the patients.

### Gray matter volume

**Figure 2:**
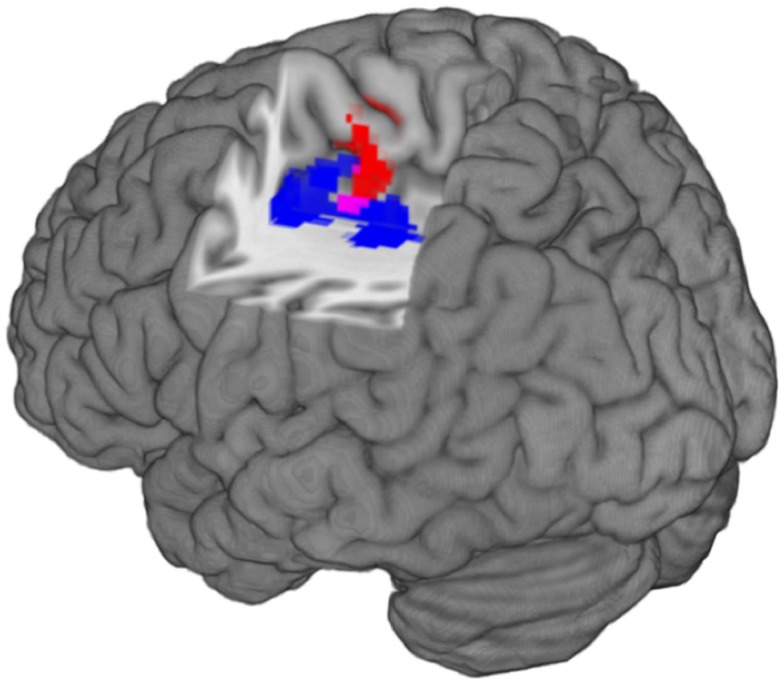
**The image shows the localization of structural and functional imaging findings in the left caudal cingulate motor**. Left is left in the image. The red cluster represents increased functional activation in patients compared to controls (*Z* > 2.3, *p* = 0.05, corrected). The blue cluster represents gray matter volume decreases in patients compared to controls (*T* > 2.5, *p* = 0.05, corrected). The purple voxels represent the overlap between function and structural findings.

In our comparison of left CMA gray matter volume in patients and controls, we observed one cluster of decreased gray matter volume in the patient group that encompassed the left caudal CMA (cluster size: 941 voxels/3176 mm^3^; *T*_CMA_ = 6.79; *p* = 0.01, corrected at the cluster level; peak voxel: *x* = −9, *y* = 4.5, *z* = 33; Figures 1 and 2).

**Figure 3:**
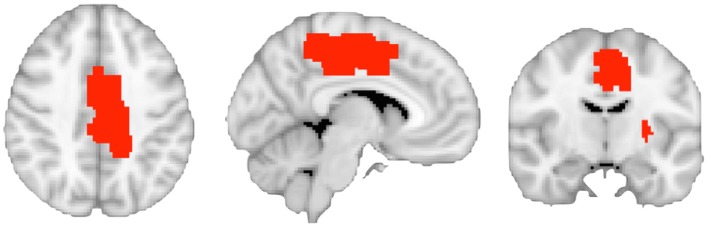
**The image shows the binarized region of interest masks defining the left caudal cingulate motor area and the left putamen regions connected to caudal motor regions in the frontal lobe**.

### Cross-modality relationships

We found a statistically significant and negative correlation (rho: −0.57, *p* = 0.002; Table 3) between mean functional activation (356 voxels/2848 mm^3^) and gray matter volume (941 voxels/3176 mm^3^) in the left caudal CMA across both groups. There was also a statistically significant and positive correlation (rho: 0.52, *p* = 0.006, Table 3) between gray matter volume (941 voxels/3176 mm^3^) and functional coupling of the left caudal CMA and left posterior putamen regions connected to caudal motor regions in the frontal lobe (31 voxels/248 mm^3^) across both groups. The spatial overlap of between-group differences in gray matter volume and functional activation is shown in Figure 2.

**Table 3:** **Non-parametric correlations between neuroimaging modalities and clinical ratings**.

Variables	Size	#Voxels (mm^3^)	Rho	*p*
Functional activation – CORE (patients)	*n* = 13	356 (2848)	0.28	0.35
Functional coupling – CORE (patients)	*n* = 13	31 (248)	−0.57	0.04
Gray matter volume – CORE (patients)	*n* = 13	941 (3176)	−0.11	0.71
Gray matter volume – Functional activation	*n* = 26	941 (3176)/356 (2848)	−0.57	0.002
Gray matter volume – Functional coupling	*n* = 26	941 (3176)/31 (248)	0.52	0.006

### Clinical correlations

We found a statistically significant and negative correlation (rho = −0.57; *p* = 0.04, Table 3) between clinical ratings of psychomotor disturbances (CORE) in patients and functional coupling of the left caudal CMA and left posterior putamen regions connected to caudal motor regions in the frontal lobe (356 voxels/2848 mm^3^). We did not find a significant correlation (rho = 0.39; *p* = 0.18) between CORE ratings and functional activation of the left caudal CMA (356 voxels/2848 mm^3^) or between CORE ratings and gray matter volume in the left caudal CMA (rho = −0.11; *p* = 0.71; 941 voxels/3176 mm^3^).

## Discussion

In the present study, we used structural and functional neuroimaging to investigate the contribution of the CMA to psychomotor disturbances in major depressive disorder. By combining neuroimaging modalities, we found converging evidence supporting a neural model of psychomotor disturbances that involves a fronto-striatal network contributing to motor execution. Specifically, we found that (1) patients activated the CMA more than healthy controls during right-handed self-paced finger-tapping; (2) functional coupling of the CMA and putamen was absent in patients compared to controls during task performance; (3) patients had decreased gray matter volume in the CMA; (4) clinical ratings of psychomotor disturbance in patients were negatively correlated with functional coupling of the CMA and putamen; and (5) gray matter volume of the CMA region with between-group differences was negatively correlated with functional activation, and positively correlated with functional coupling.

Our study lends experimental support to the involvement of a fronto-striatal network in the emergence of psychomotor disturbances in major depressive disorder. In our study, gray matter volume decreases were associated with decreased functional coupling of the left caudal CMA and left posterior putamen during motor execution of a right-handed self-paced finger-tapping task. The midcingulate region comprising the CMA is known to provide extensive output to skeletomotor areas, including the striatum (41). Changes in the CMA would, therefore, impact upon its glutamatergic inputs to subcortical motor systems and, in turn, lead to impairments in motor behavior, i.e., psychomotor disturbances. Although beyond the scope of the current study, this finding may be relevant to neuron loss in cortical layer V, from which efferent fibers are derived, in accordance with Vogt’s model of motor dysfunction (50).

It is interesting to note the relative position of the clusters resulting from the functional and the structural analysis: few voxels show an overlap between the functional cluster representing increased neural activity and the structural cluster representing decreased gray matter volume in the CMA of patients (Figure 2). The interpretation of the spatial extent of clusters, resulting from cluster-based statistics is known to be problematic, especially when relatively low cluster-forming thresholds are used like in this study (i.e., *T* > 2.5) (68). Nevertheless, the increase of functional activation in an area that seems to be spared by gray matter volume loss may represent a less affected region or a compensatory mechanism. Compensatory cortical plasticity has been previously described after acute brain lesions but a similar mechanism could also be involved following more chronic alterations of brain networks in the context of mental illness (69).

This study conforms to previous studies that have shown paralimbic hyperactivation in major depressive disorder using both molecular metabolic imaging and functional magnetic resonance imaging with BOLD (70, 71). Hypothetically, a loss of neurons in cingulate layer V suggested by Vogt et al. (50) could arise from excess glutamate signaling with impaired plasticity and neural resilience (72). However, the BOLD signal comprises several neuronal and vascular factors that preclude a specific association of increased activation and hyperglutamatergia (73). Indirect correlational evidence for cingulate hyperglutamatergia may be inferred from the positive correlation of glutamate signaling and spontaneous neural activity of paralimbic medial frontal and posterior cingulate regions: the so-called “default-mode” network (74, 75). Several studies suggest that this network is hyperactivated in major depressive disorder (76–78). In a previous study of the bipolar disorder subgroup of this sample (*n* = 9), there was an increased activation among depressed patients in the default-mode network during motor execution (15). However, the opposite association between glutamate, functional connectivity, and depression severity has also been shown (79). Thus, we remain cautious regarding definitive interpretations of our findings in the context of the pathophysiology underlying psychomotor disturbances in major depressive disorder.

Our study has limitations. First, this is a ROI study where we had an *a priori* hypothesis involving selected brain regions in the left hemisphere known to activate during simple right-hand movements. The disadvantage of the ROI approach is that it discerns a comprehensive observation of the full large-scale neuronal networks implicated in these disturbances (80). However, our motivation for choosing the ROI approach was that our hypothesis addressed specific ideas based on previous research on the CMA in major depressive disorder. An advantage of the ROI approach is that it potentially increases the sensitivity of the neuroimaging methods we applied, and previous research suggests that ROI analyses may provide a better statistical control of both type I and type II errors than in whole-brain analyses as the ROI approach is less dependent on correction for multiple comparisons (81). This becomes even more important in smaller samples such as ours. Second, our brain imaging approach is restricted to inference from indirect monitoring of the disease underlying major depressive disorder. Functional imaging studies and anatomical mapping advocate a limbic hyperactivation in major depressive disorder and suggest that more anterior cingulate cortex regions convey limbic signals to the CMA, which in turn suggests that our observed CMA alterations may only be a tertiary marker for a disease that alters upstream nodes in the same neural network (29, 43). The BOLD contrast mechanism itself also highlights the importance of inputs to CMA (82). Third, the CMA and putamen are both rich in ascending midbrain dopamine afferents that contribute to the initiation, preparation, execution, and control of movement (56, 83). Six patients in our sample were treated with antipsychotic medication that affects dopamine transmission in both regions. This could potentially lead to misrepresentative and biased results. However, neuroimaging metrics in these six individuals were distributed across the whole patient sample, and there were no outliers in the patient group with respect to functional activation, gray matter volume, or functional coupling. Fourth, there are many types of motor behavior, and it is possible that finger-tapping does not engage identical fronto-striatal networks in the same way as more elaborate motor behavior. The strength of the finger-tapping task is that it is sensitive to pathology in fronto-striatal neurocircuitry and is considered to measure a defined entity: motor speed (84). Fifth, the BOLD signal in the CMA may be subject to noise due to partial volume effects because of the spatial resolution of our functional scans. This contributes to a possible source of error in our PPI analysis. Finally, given the small sample size of our groups, the GLM may not provide the best fit of our second-level data. However, we analyzed our data at second level using a mixed effects analysis (FMRIB’s Local Analysis of Mixed Effects stage 1 and stage 2) similar to a vast majority of comparable functional imaging studies. In order to confirm the validity of our results, we also re-analyzed data at second level with Randomise for non-parametric inference while using an identical statistical threshold. The spatial extent of those statistically significant results was close to identical regarding the CMA cluster of between-group differences. We would like to reiterate that even if the GLM does not provide the best fit for our second level analysis, the corrected *p*-value calculated by Randomise is valid, regardless the distribution of our data.

## Conclusion

Our data are cross-sectional but implicates the possibility of a chain of events where primary neural hyperactivation in paralimbic regions has secondary effects on gray matter volume in fronto-striatal networks involved in movement. In our study, functional activation was negatively correlated with gray matter volume, which suggests an association between paralimbic hyperactivation and gray matter alterations. The positive correlation between gray matter volume and functional coupling supports the notion of diminished cortical output via layer V to subcortical regions that are involved in motor control. The functional coupling was also negatively correlated with clinical ratings, which suggests a role of decreased functional connectivity within fronto-striatal networks in the emergence of clinical psychomotor disturbances in major depressive disorder. Thus, our results propose an involvement of the CMA in the generation of psychomotor disturbances in a major depressive disorder. Our data also suggest the possibility of elucidating the causality of these events by using an experimental longitudinal study design and analyses of directional effective connectivity within these networks during motor performance.

## Author Contributions

All authors made substantial contributions to the conception and design of this work. Benny Liberg and Mats Adler collected the original data. Benny Liberg, Paul Klauser, and Ian H. Harding performed the analyses. All authors contributed to the interpretation of data for this work. Benny Liberg, Paul Klauser, and Ian H. Harding drafted the manuscript, which all authors critically revised for important intellectual content. All authors approved the final version of the manuscript. All authors agree to be accountable for all aspects of the work in ensuring that questions related to the accuracy or integrity of any part of the work are appropriately investigated and resolved.

## Conflict of Interest Statement

The authors declare that the research was conducted in the absence of any commercial or financial relationships that could be construed as a potential conflict of interest.
